# Socioeconomic inequalities in adolescent mental health in the Nordic countries in the 2000s - A study using cross-sectional data from the Health Behaviour in School-aged Children study

**DOI:** 10.1186/s13690-024-01240-5

**Published:** 2024-02-07

**Authors:** Maria Corell, Peter Friberg, Max Petzold, Petra Löfstedt

**Affiliations:** 1 School of Public Health and Community Medicine, Institute of Medicine, Gothenburg University, 405 30 Göteborg, Box 428, Sweden; 2 Public Health Agency of Sweden, 171 82 Solna, Sweden

**Keywords:** Adolescent health, Mental health, Subjective health complaints, Life satisfaction, Income inequality, At-risk-of-poverty rate, Socioeconomic inequalities, Social gradient, Nordic countries

## Abstract

**Background:**

Adolescents in Sweden experience more mental health problems and lower mental well-being than adolescents in other Nordic countries. According to the literature, one possible explanation may be differences in income inequality. The at-risk-of-poverty rate varies significantly across the Nordic countries, and the highest rate is found in Sweden. The aims of the study were to examine socioeconomic inequalities in subjective health complaints and life satisfaction among adolescents in the Nordic countries during 2002 − 2018 and to explore whether subjective health complaints and life satisfaction were related to income inequality in terms of the at-risk-of-poverty rate at the country level.

**Methods:**

Data regarding 15-year-olds from the Health Behaviour in School-aged Children study from five survey rounds (2002 − 2018) were used (*n* = 41,148). The HBSC Symptoms Checklist and Cantril’s ladder were used as measures of subjective health complaints and life satisfaction, respectively. The Family Affluence Scale, the Perceived Family Wealth item and the at-risk-of-poverty rate in each country were used as measures of individual-level socioeconomic conditions and country-level income inequality. Statistical methods involved ANOVA, multiple linear regressions and multilevel regression analyses.

**Results:**

Absolute and relative socioeconomic inequalities in both subjective health complaints and life satisfaction were found in all countries. Sweden showed average socioeconomic inequalities, Iceland the largest and Denmark the smallest. Country-level income inequality in terms of the at-risk-of-poverty rate was associated with a higher prevalence of subjective health complaints and lower levels of life satisfaction in all countries.

**Conclusion:**

Socioeconomic inequalities in adolescent mental health and well-being persisted in Nordic countries in the 2000s. Increasing income inequality may have contributed to higher levels of SHC and lower LS in Sweden compared to the other Nordic countries. Policies improving families’ socioeconomic conditions and reducing income inequality at the country level are needed to improve and reduce inequalities in mental health and well-being among adolescents.

**Supplementary Information:**

The online version contains supplementary material available at 10.1186/s13690-024-01240-5.

**Table:** 

**Text box 1. Contributions to the literature**
• The study examines the role of absolute and relative socioeconomic conditions and income inequality in adolescent mental health and well-being in Nordic countries during the last two decades.
• In contrast to most previous studies addressing the association between income inequality and adolescent health, which have used the Gini coefficient, this study has used the at-risk-of-poverty rate.
• The study suggests that the at-risk-of-poverty rate may be used as an alternative measure of income inequality among countries where income inequality in terms of the Gini may be of similar magnitude.

## Background

In recent decades, self-reported mental health problems have increased among children and adolescents in many countries, including the Nordic countries [1, 2]. In Sweden, the proportion of 15-year-old girls and boys reporting multiple subjective health complaints has more than doubled since the mid-1980s [3]. In 2021/22, 77% of girls and 46% of boys reported multiple subjective health complaints [3]. In contrast, life satisfaction (LS) measured with Cantril’s ladder ranging from 0 to 10 was rather stable among both 15-year-old Swedish girls (6.4 − 7.0) and boys (7.2 − 7.7) during the 2000s [3]. Furthermore, the proportion reporting multiple subjective health complaints was higher and the mean values of LS were lower among 15-year-old girls and boys in Sweden than in all other Nordic countries according to the most recent available international data (2018) [4]. Together, these two indicators of mental health and well-being [5] suggest that adolescents in Sweden experience more mental health problems and lower mental well-being than adolescents in other Nordic countries.

Several explanations for the gradual increase in mental health problems among adolescents in Sweden during recent decades have been proposed, such as the extensive school reforms that have taken place since the 1990s, which have resulted in increased school pressure and worse school performance [6, 7]. Another explanation is higher educational demands in the labour market [7, 8]. The role of various societal changes, such as increasing income inequality, has been less explored, although income inequality has increased in most OECD countries since the mid-1980s, especially in the Nordic countries [9].

Sweden has experienced the largest increase in income inequality among the Nordic countries and has the highest income inequality in terms of the Gini coefficient and the at-risk-of-poverty rate [10, 11]. The Gini coefficient, ranging from 0 to 1, varies between 0.261 in Iceland (2017) and 0.289 in Sweden (2020) [10]. The at-risk-of-poverty rate is the proportion of the population whose equivalised disposable income is below 60% of the national median equivalised disposable income after social transfers [11]. The rate varies significantly from 8.8% in Iceland (2018) to 16.4% in Sweden (2018) [11]. The Gini coefficient and the at-risk-of-poverty rate are highly correlated [12]. Regardless of which measure of income inequality is used, the trends often look similar when the same income measures are used [9]. Individuals’ yearly income (earnings or total market incomes) before or after taxes and transfers and adjusted to household size (equivalised) are often used as measures of income.

Despite increased income inequality, both the Gini coefficient and the at-risk-of-poverty rate remain low in the Nordic countries compared to most other European countries [10, 11]. The Nordic countries share cultural, historical and political heritage and are characterized by universal and generous benefits, redistribution and a commitment to full employment and income protection (belong to the “social democratic welfare regime” according to Esping-Andersen, 1990 [13]). Nevertheless, we are interested in exploring the role of increased income inequality for adolescents’ mental health in the Nordic countries.

## Theoretical framework

Apart from biological factors, such as age, sex and constitutional factors, the mental health of individuals is shaped by the social, environmental and economic conditions in which they are born, grow up, work and age [14]. Inequalities in mental health are mainly caused by structural differences in different socioeconomic groups’ access to social, economic and political resources, which affect health through different environmental, psychological and behavioural processes. With each step one moves up the social ladder, in terms of education, social class or income, the better one’s health (known as the social gradient in health) [15]. Inequalities in health emerge early in life and become more pronounced throughout childhood [16].

Although income is only one dimension of socioeconomic status, it is largely influenced by education and closely related to occupation [15]. Therefore, the the three prevailing hypotheses explaining the effect of income and income inequality on health can also be understood as explaining the effect of socioeconomic status in general on health. The three hypotheses are described below:

The *absolute-income hypothesis* (AIH), also known as the *materialist* or *structuralist explanation*, states that income has a direct effect on the health of an individual, as it enables housing, food, physical exercise and medical care. Occupation is closely related to income, and the work environment and occupational hazards also have a direct effect on health [15].

The *relative-income hypothesis* (RIH), also known as the *psychosocial theory*, states that the income of others affects the health of an individual through psychosocial processes involving social participation and opportunities to control life circumstances [15]. Relative deprivation creates stress and shame and reduces the health of an individual. In rich countries, where absolute deprivation is low, the social gradient shifts to relative deprivation [17].

The *income-inequality hypothesis* (IIH) states that income inequality has a detrimental effect on all individuals’ health in society. Income inequality is negatively associated with trust and social capital and higher crime and accident rates. It is also associated with morbidity, mortality and mental illness [17]. Income inequality is related to the social gradient in health: in more egalitarian societies, the burden of relative deprivation is reduced, hence decreasing social inequalities in health [17].

The three hypotheses are valid for children and adolescents too. First, economic resources enable parents to invest in their children’s development and thereby foster their social, emotional and cognitive well-being [18]. Second, even young children are aware of social status and make social comparisons, affecting their health [19]. Family income may also affect children and adolescent indirectly, for instance family financial difficulties may affect the relationship between parents and children negatively [18].

### Previous research

There is vast research on socioeconomic inequalities in health among children and adolescents. A systematic overview has shown a consistent, negative association between socioeconomic status, such as parental education, social class and income, and mental health problems among children and adolescents 4–18 years of age [20].

Previous research based on data from the Health Behaviour in School-aged Children (HBSC) study, using the Family Affluence Scale as the basis of both absolute and relative measures of socioeconomic status, has shown that there are both absolute and relative socioeconomic inequalities in adolescents’ subjective health complaints [21–24] and life satisfaction [24, 25]. Thus, previous research supports both the absolute-income and the relative-income hypotheses.

Research based on HBSC data together with macro-level indicators has also shown that higher income inequality in terms of Gini is associated with a higher prevalence of subjective health complaints [21, 24, 26, 27] and lower life satisfaction [24, 28] among children and adolescents at a national level. Higher income inequality is also associated with larger socioeconomic inequalities in subjective health complaints [24, 27] and life satisfaction [24, 25, 28] among children and adolescents. These findings support the income-inequality hypothesis.

Further, income inequality and the at-risk-of-poverty rate were strongly associated to the Unicef index of child health and well-being in rich countries [19]. Countries with social democratic regimes, higher public spending, and lower income inequalities have populations with better health [29].

To our knowledge, no study has addressed the role of absolute and relative socioeconomic conditions and the role of income inequality in mental health and well-being among adolescents in the Nordic countries during the 2000s. Nor have we found a study using the at-risk-of-poverty rate at the country level as a measure of income inequality when addressing its association with mental health and well-being.

### Aims

The aims of the study were (i) to examine individual-level socioeconomic inequalities in SHC and LS among adolescents in the Nordic countries in the 2000s and (ii) to explore whether SHC and LS were related to income inequality at the country level.

The following research questions were addressed:

i. Were there individual-level absolute and relative socioeconomic inequalities in SHC and LS among adolescents in the Nordic countries from 2002 − 2018?
ii. Was income inequality in terms of the at-risk-of-poverty rate at the country level related to the prevalence of SHC and levels of LS among adolescents in the Nordic countries 2002 − 2018?

The analyses were performed for girls and boys together and separately.

## Methods

### Data

Data from the Health Behaviour in School-aged Children (HBSC) study in Sweden, Norway, Finland, Denmark and Iceland were used. The HBSC study is a World Health Organization collaborative cross-sectional study currently conducted in 50 countries across Europe and North America. The study is carried out every four years in accordance with a common research protocol [5]. The survey was completed anonymously during school hours. Data from five surveys (2002, 2006, 2010, 2014 and 2018) regarding 15-year-olds were used (*n* = 41,148). See Supplementary Table 1 for the number of participants in each country from 2002 − 2018. Note that Iceland joined the study in 2006. Data from Eurostat regarding the at-risk-of-poverty rate were also used.

### Variables

*Life satisfaction (LS)* was measured with Cantril’s ladder [30]. The adolescents were asked to rate their life satisfaction using a visual analogue scale with 11 steps: the top indicates the best possible life, and the bottom the worst. In this study, the variable was used as a continuous variable (from 0 to 10). The scale has been adopted for children and has shown good reliability and good convergent validity with several measures of health and well-being [31].

*Subjective Health Complaints (SHCs)* were measured with the HBSC Symptoms Checklist (HBSC-SCL). Adolescents were asked how often they had experienced the following symptoms in the last six months: headache; stomach ache; backache; feeling low; irritability or bad temper; feeling nervous; difficulties in getting to sleep; and feeling dizzy. Response options for each symptom ranged from “about every day” to “rarely or never”, which were reversely coded (1 = rarely or never, 2 = about every month, 3 = about every week, 4 = more than once a week and 5 = about every day) The answers were summed for children who had answered at least seven of the items (7 − 40). Cronbach’s alpha in our sample was 0.835. In this study, the variable was used as a continuous variable (ranging from 7 to 40, where higher values indicated more complaints). The sum score of the eight health complaints reflects the total symptom load among adolescents and has previously been used [32–34]. The instrument has been validated several times (see [5] for an overview).

The *Family Affluence Scale (FAS)* was used as a measure of absolute socioeconomic status. The scale was developed in the 1990s to capture the family’s socioeconomic conditions and has been revised several times as lifestyle has changed and living conditions have improved [35]. In the years 2002 − 2010, it consisted of four items (known as the FAS II) regarding the family’s material assets: number of cars (0, 1, 2 or more), number of computers (0, 1, 2, > 2), unshared bedroom (no/yes), and the number of holidays during the last 12 months (0, 1, 2, 3 or more). From 2014 − 2018, the scale consisted of two more items (known as FAS III): dishwasher (no/yes) and number of bathrooms (0, 1, 2, > 2). Additionally, the holiday item was changed to holidays *abroad*. The children’s answers to the items were summed. For each country, age-group and gender-specific ridit scores were calculated to identify groups of children in the lowest 20% (low affluence), middle 60% (medium affluence) and highest 20% (high affluence) in accordance with current guidelines [5]. See Supplementary Table 2. The FAS II was used as a continuous variable (0–9) in the regression analyses. The scale has been validated several times, most recently by Corell et al., 2021 [35], who showed that the FAS III may be used as an alternative measure of parental earned income in studies using self-reported socioeconomic status among adolescents, and Torsheim et al., 2016 [36], who showed that FAS III correlated with parental reported income groups in six out of eight European countries.

*Perceived family wealth (PFW)* was used as a measure of relative socioeconomic status and measured with the question “How well off do you think your family is?”, with the response options “very well off”, “quite well off”, “average”, “not so well off”, and “not at all well off”. The variable was both used as a continuous variable (1 − 5), where responses were reversed from 1 (not at all well off) to 5 (very well off), and as a categorical variable (not well off, average and well off). The instrument was mandatory for all countries participating in the HBSC study 2002 − 2014 but optional in 2018. Denmark and Iceland did not include the item in 2018. See Supplementary Table 3.

*The at-risk-of-poverty rate* was used as a measure of income inequality. The rate is the proportion of the population whose equivalised disposable income is below 60% of the national median equivalised disposable income after social transfers. Data came from official statistics from Eurostat and concerned the whole population, regardless of age. Because HBSC data collections took place in autumn and winter during each survey, mean values were calculated for 2001 and 2002, 2005 and 2006, etc. See Supplementary Table 4.

### Statistical methods

Within-country individual-level socioeconomic inequalities in SHC and LS were addressed by calculations of mean values for each FAS group and PFW group from 2002 − 2018. ANOVA with Bonferroni correction was performed to determine whether differences in mean values across FAS and PFW groups were statistically significant (*p* < 0.05). Calculations were made for boys and girls together and separately (results not shown, only statistically significant differences between girls and boys are presented under Results).

Between-country comparisons of individual-level socioeconomic inequalities in SHC and LS were addressed by performing multiple linear regressions for each country. The relationships between SHC and LS, FAS/PFW, survey year and gender in each country were examined with unstandardized B coefficients and *p* values. To determine whether the B coefficients were significantly different from each other, their 95% confidence intervals were estimated. In the event that they did not overlap, the B coefficients were considered significantly different from each other.

To examine the relation between the at-risk-of-poverty rate and SHC/LS, multilevel regression analyses were performed. First, empty models with SHC/LS were performed to determine the level of clustering of children within countries, resulting in intraclass correlation coefficients (ICCs). In the second model, the relation between SHC/LS and survey year and the at-risk-of-poverty rate was examined. In the third model, individual-level variables (gender, FAS II and PFW) were added. Random intercepts for each country and fixed effects for all independent variables were applied in the second and third models.

All analyses were performed for girls and boys together and separately. All analyses were performed in IBM SPSS v. 26.0.

## Results

### Individual-level socioeconomic inequalities in subjective health complaints in each Nordic country

First, we examined mean values of SHC across the FAS groups (Table 1). In Sweden, Finland and Denmark, levels of SHC were higher in the low FAS group than in the medium FAS group in individual years. In Norway, differences in SHC across FAS groups were observed at the beginning of the period (2002 − 2010). In contrast, significant differences in SHC across FAS groups were found every year (2006 − 2018) in Iceland.

**Table 1:** The distribution of SHC across FAS groups and associations between FAS and SHC in each Nordic country, 2002–2018

Year		Sweden		Norway		Finland		Denmark		Iceland	
*Descriptive statistics*		Mean	*n*	Mean	*n*	Mean	*n*	Mean	*n*	Mean	*n*
2002	Low FAS	18.9^m^	*289*	17.1^mh^	*251*	18.1^m^	*303*	14.6	*256*		
	Medium FAS	17.7^l^	*711*	15.3^l^	*961*	17.2^l^	*1048*	14.8	*815*		
	High FAS (ref)	17.9	*195*	15.0	*379*	17.0	*355*	14.9	*263*		
	Total	18.1	*1203*	15.5	*1603*	17.3	*1732*	14.8	*1353*		
2006	Low FAS	19.5^m^	*260*	16.7^m^	*310*	18.2	*213*	15.5	*270*	20.0^mh^	*340*
	Medium FAS	18.4^l^	*887*	15.6^l^	*941*	17.7	*1107*	15.2	*870*	18.4^l^	*1156*
	High FAS (ref)	18.6	*339*	16.0	*238*	18.2	*318*	15.1	*334*	17.7	*291*
	Total	18.6	*1512*	15.9	*1501*	17.8	*1675*	15.3	*1536*	18.6	*1873*
2010	Low FAS	17.9	*403*	18.9^mh^	*228*	17.7	*349*	16.2^m^	*240*	19.6^mh^	*512*
	Medium FAS	18.3	*1078*	15.9^l^	*765*	17.3	*1304*	14.9^l^	*803*	17.6^l^	*2254*
	High FAS (ref)	17.5	*525*	16.2	*324*	17.1	*397*	15.1	*151*	17.5	*811*
	Total	18.0	*2066*	16.5	*1328*	17.4	*2104*	15.2	*1219*	17.9	*3640*
2014	Low FAS	19.9	*437*	16.5	*136*	16.7	*254*	17.0	*184*	19.1^m^	*775*
	Medium FAS	19.0	*1503*	16,7	*578*	16.0	*1244*	16.4	*817*	18.3^l^	*1623*
	High FAS (ref)	19.0	*677*	15.9	*158*	15.7	*383*	16.2	*168*	18.2	*671*
	Total	19.1	*2730*	16.5	*909*	16.0	*1954*	16.4	*1251*	18.4	*3300*
2018	Low FAS	20.1	*224*	17.3	*113*	19.3	*222*	17.3^m^	*163*	20.1^mh^	*318*
	Medium FAS	20.0	*1081*	16.8	*428*	18.7	*604*	15.9^l^	*407*	18.7^lh^	*1301*
	High FAS (ref)	19.5	*228*	17.9	*110*	18.2	*191*	16.6	*125*	17.5	*504*
	Total	19.9	*1571*	17.1	*667*	18.8	*1068*	16.5	*757*	18.6	*2157*
**Linear regressions**		**B**	**95% C.I.**	**B**	**95% C.I.**	**B**	**95% C.I.**	**B**	**95% C.I.**	**B**	**95% C.I.**
	FAS II (0–7)	-0.18***	-0.26 to -0.10	-0.27***	-0.36 to -0.17	-0.13***	-0.21 to -0.05	-0.14***	-0.22 to -0.05	-0.49***	-0.58 to -0.41
	Survey year	0.12***	0.09 to 0.14	0.11***	0.08 to 0.14	0.00	-0.02 to 0.03	0.12***	0.10 to 0.15	0.00	-0.03 to 0.03
	Girl	4.28***	4.01 to 4.54	3.30***	3.00 to 3.60	3.17***	2.92 to 3.42	2.73***	2.46 to 3.00	3.70***	3.43 to 3.96
	Adjusted R	0.112		0.086		0.071		0.074		0.078	
	F	373.2	0.000	187.1	0.000	212.0	0.000	158.1	0.000	294.6	0.000

In the descriptive statistics: Significant (*p* < 0.05) test results between FAS groups: ^l^= compared to the low FAS group ^m^=compared to the medium FAS group. ^h^=compared to the high FAS group

In the regression models: * *p* < 0.05 ** *p* < 0.01 ***p < 0.001. *P*-values adjusted for multiple testing

The multiple linear regressions showed that in all five countries, higher levels of FAS were significantly associated with lower levels of SHC (Table 1). The regressions also showed that FAS was more strongly related to SHC in Iceland (B = -0.49) than in the other countries.

Second, we examined the mean values of SHC across PFW groups and found significant differences in all countries every year (2002 − 2018) (Table 2). With the exception of Denmark, SHC decreased significantly between the “not well off” and “average” PFW groups and between the “average” and “well off” groups in all countries in nearly all years. In Denmark, levels of SHC were higher in the “not well off” group than in the other two groups, but no significant differences in SHC between adolescents in the “average” and “well off” groups were found. In Denmark, socioeconomic inequalities in SHC were mainly found among girls and not among boys. In Norway, socioeconomic inequalities were larger among girls than boys.

**Table 2:** The distribution of SHC across PFW groups and associations between PFW and SHC in each Nordic country, 2002–2018

Year		Sweden		Norway		Finland		Denmark		Iceland	
*Descriptive statistics*		Mean	*n*	Mean	*n*	Mean	*n*	Mean	*n*	Mean	*n*
2002	Not well off	21.4^aw^	*110*	18.8^aw^	*136*	19.8^aw^	*118*	16.9^aw^	*42*		
	Average	19.3^nw^	*306*	15.8^nw^	*556*	17.7^nw^	*561*	14.8^n^	*1068*		
	Well off (ref)	17.1	*777*	14.8	*902*	16.8	*1038*	14.4	*230*		
	Total	18.1	*1203*	15.5	*1603*	17.3	*1732*	14.8	*1353*		
2006	Not well off	24.0^aw^	*100*	20.1^aw^	*77*	20.4^w^	*72*	17.8^aw^	*55*	24.0^aw^	*85*
	Average	18.8^n^	*291*	16.5^nw^	*383*	18.6^w^	*517*	15.3^n^	*1093*	19.8^nw^	*502*
	Well off (ref)	18.1	*1110*	15.2	*1012*	17.3	*1073*	14.9	*340*	17.8	*1268*
	Total	18.6	*1512*	15.9	*1501*	17.8	*1675*	15.3	*1536*	18.6	*1873*
2010	Not well off	23.1^aw^	*115*	22.5^aw^	*70*	21.2^aw^	*148*	18.7^aw^	*44*	23.7^aw^	*218*
	Average	18.9^nw^	*432*	17.4^nw^	*294*	18.6^nw^	*599*	15.1^n^	*906*	18.8^nw^	*1039*
	Well off (ref)	17.3	*1472*	15.6	*811*	16.4	*1343*	15.0	*238*	16.8	*1950*
	Total	18.0	*2066*	16.5	*1328*	17.4	*2104*	15.2	*1219*	17.9	*3640*
2014	Not well off	23.1^aw^	*141*	22.1^aw^	*32*	19.4^aw^	*182*	19.4^aw^	*60*	24.3^aw^	*187*
	Average	20.6^nw^	*562*	18.2^nw^	*210*	16.5^nw^	*558*	16.3^n^	*870*	19.8^nw^	*999*
	Well off (ref)	18.5	*1971*	15.7	*647*	15.2	*1205*	15.9	*301*	17.2	*2065*
	Total	19.1	*2730*	16.5	*909*	16.0	*1954*	16.4	*1251*	18.4	*3300*
2018	Not well off	24.5^aw^	*49*	20.3^w^	*22*	25.5^aw^	*63*				
	Average	21.4^nw^	*312*	19.2^w^	*116*	20.4^nw^	*227*				
	Well off (ref)	19.4	*1187*	16.3	*492*	17.7	*757*				
	Total	19.9	*1571*	17.1	*667*	18.8	*1068*				
**Regression analysis**		**B**	**95% C.I.**	**B**	**95% C.I.**	**B**	**95% C.I.**	**B**	**95% C.I.**	**B**	**95% C.I.**
	PFW (1–5)	-1.43***	-1.57 to -1.29	-1.33***	-1.52 to -1.15	-1.13***	-1.26 to -0.99	-0.38**	-0.64 to -0.11	-1.69***	-1.86 to -1.53
	Survey year	0.13***	0.10 to 0.15	0.12***	0.09 to 0.15	0.00	-0.03 to 0.02	0.11***	0.08 to 0.14	-0.02	-0.07 to 0.03
	Girl	4.21***	3.96 to 4.47	3.14***	2.84 to 3.43	2.95***	2.70 to 3.20	2.68***	2.39 to 2.96	3.46***	3.18 to 3.75
	Adjusted R	0.150		0.112		0.097		0.071		0.112	
	F	524.9	0.000	244.1	0.000	303.6	0.000	133.8	0.000	351.4	0.000

In the descriptive statistics: Significant (*p* < 0.05) test results between PFW groups: ^w^= compared to the well off group ^a^=compared to the average group. ^n^=compared to the not well off group

In the regression models: * *p* < 0.05 ** *p* < 0.01 ***p < 0.001. *P*-values adjusted for multiple testing

The multiple linear regressions showed that in all countries, higher levels of PFW were significantly associated with lower levels of SHC (Table 2). The relation between PFW and SHC was stronger in Iceland (B = -1.69) than in the other countries, except Sweden.

The regression analyses also revealed that SHC increased over time in three countries (Sweden, Norway and Denmark) (Tables 1 and 2). They also showed that levels of SHC were higher among girls than boys in all countries.

### Individual-level socioeconomic inequalities in life satisfaction in each Nordic country

Next, we examined mean values of LS across the FAS groups (Table 3). Generally, differences in LS across FAS groups were found in all countries for the entire period. In Denmark, socioeconomic differences across FAS groups were more pronounced among girls than boys in most years.

**Table 3:** The distribution of LS across FAS groups and associations between FAS and LS in each Nordic country, 2002–2018

Year		Sweden		Norway		Finland		Denmark		Iceland	
*Descriptive statistics*		Mean	*n*	Mean	*n*	Mean	*n*	Mean	*n*	Mean	*n*
2002	Low FAS	6.7^mh^	*288*	6.4^mh^	*249*	7.1^mh^	*302*	7.5^h^	*259*		
	Medium FAS	7.2^l^	*713*	7.1^l^	*965*	7.6^lh^	*1045*	7.6	*809*		
	High FAS (ref)	7.2	*196*	7.3	*383*	8.0	*353*	7.9	*264*		
	Total	7.1	*1204*	7.0	*1609*	7.6	*1729*	7.7	*1349*		
2006	Low FAS	7.0^h^	*257*	7.0^mh^	*307*	7.4^h^	*214*	7.3^h^	*268*	6.9^mh^	*336*
	Medium FAS	7.3^h^	*884*	7.6^l^	*947*	7.6	*1095*	7.6	*862*	7.5^lh^	*1147*
	High FAS (ref)	7.6	*338*	7.6	*234*	7.8	*317*	7.8	*331*	7.9	*286*
	Total	7.3	*1502*	7.5	*1503*	7.6	*1660*	7.6	*1524*	7.4	*1849*
2010	Low FAS	6.9^h^	*395*	6.7^mh^	*225*	7.2^mh^	*348*	6.9^mh^	*242*	6.7^mh^	*513*
	Medium FAS	7.1^h^	*1054*	7.5^l^	*754*	7.5^lh^	*1294*	7.6^l^	*804*	7.6^lh^	*2247*
	High FAS (ref)	7.5	*515*	7.8	*324*	7.9	*393*	7.6	*150*	7.9	*807*
	Total	7.2	*2024*	7.4	*1314*	7.5	*2089*	7.5	*1220*	7.5	*3630*
2014	Low FAS	6.4^mh^	*433*	7.3^h^	*140*	7.1^mh^	*261*	7.0^mh^	*193*	7.2^mh^	*755*
	Medium FAS	6.8^lh^	*1483*	7.5	*597*	7.5^lh^	*1262*	7.4^l^	*843*	7.5^lh^	*1544*
	High FAS (ref)	7.1	*669*	7.8	*159*	7.8	*393*	7.6	*170*	7.8	*657*
	Total	6.8	*2679*	7.5	*910*	7.5	*1960*	7.4	*1263*	7.5	*3275*
2018	Low FAS	6.6^mh^	*215*	6.9^mh^	*112*	7.4	*221*	7.1^mh^	*164*	6.9^mh^	*320*
	Medium FAS	7.1^lh^	*1070*	7.5^l^	*426*	7.5	*597*	7.6^l^	*408*	7.2^lh^	*1300*
	High FAS (ref)	7.5	*225*	8.0	*110*	7.7	*190*	7.9	*125*	7.6	*506*
	Total	7.1	*1546*	7.5	*665*	7.5	*1061*	7.5	*762*	7.3	*2172*
**Linear regressions**		**B**	**95% C.I.**	**B**	**95% C.I.**	**B**	**95% C.I.**	**B**	**95% C.I.**	**B**	**95% C.I.**
	FAS II (0–9)	0.16***	0.14 to 0.18	0.20***	0.17 to 0.23	0.12***	0.10 to 0.14	0.12***	0.09 to 0.14	0.21***	0.19 to 0.24
	Survey year	-0.03***	-0.03 to -0.02	0.02***	0.01 to 0.03	-0.02***	-0.03 to -0.01	-0.03***	-0.03 to -0.02	-0.01	-0.02 to 0.00
	Girl	-0.69***	-0.76 to -0.61	-0.50***	-0.60 to -0.41	-0.35***	-0.42 to -0.28	-0.49***	-0.58 to -0.41	-0.36***	-0.43 to -0.29
	Adjusted R	0.055		0.051		0.037		0.026		0.041	
	F	170.8		108.4		77.1		76.3		149.5	

In the descriptive statistics: Significant (p < 0.05) test results between FAS groups: ^l^= compared to the low FAS group ^m^=compared to the medium FAS group. ^h^=compared to the high FAS group.

In the regression models: * p < 0.05 ** p < 0.01 ***p < 0.001. P-values adjusted for multiple testing.

The multiple linear regressions showed that there was a positive statistically significant association between FAS and LS among adolescents in all Nordic countries. The relation between FAS and LS was stronger in Norway and Iceland than in Denmark and Finland.

Finally, in all countries, there were significant differences in LS across PFW groups in all years (2002 − 2018) (Table 4). LS decreased significantly between the “not well off” and “average” PFW groups and between the “average” and “well off” groups in all countries in nearly all years.

**Table 4:** The distribution of LS across PFW groups and associations between PFW and LS in each Nordic country, 2002 − 2018

Year		Sweden		Norway		Finland		Denmark		Iceland	
*Descriptive statistics*		Mean	*n*	Mean	*n*	Mean	*n*	Mean	*n*	Mean	*n*
2002	Not well off	5.7^aw^	*107*	5.4^aw^	*135*	5.9^aw^	*118*	6.3^aw^	*41*		
	Average	6.8^nw^	*306*	6.8^nw^	*558*	7.3^nw^	*563*	7.6^nw^	*1065*		
	Well off (ref)	7.4	*781*	7.4	*906*	8.0	*1033*	8.1	*231*		
	Total	7.1	*1204*	7.0	*1609*	7.6	*1729*	7.7	*1349*		
2006	Not well off	5.4^aw^	*100*	5.8^aw^	*75*	6.8	*72*	6.6^aw^	*55*	5.4^aw^	*86*
	Average	6.9^nw^	*287*	7.0^nw^	*382*	7.3^w^	*512*	7.5^nw^	*1084*	6.9^nw^	*499*
	Well off (ref)	7.6	*1103*	7.8	*1016*	7.9	*1063*	7.8	*338*	7.8	*1249*
	Total	7.3	*1502*	7.5	*1503*	7.6	*1660*	7.6	*1524*	7.4	*1849*
2010	Not well off	5.4^aw^	*110*	5.6^aw^	*69*	6.2^aw^	*147*	6.2^aw^	*44*	5.7^aw^	*214*
	Average	6.6^nw^	*422*	7.0^nw^	*289*	7.3^nw^	*594*	7.4^nw^	*907*	7.1^nw^	*1034*
	Well off (ref)	7.5	*1443*	7.7	*808*	7.8	*1334*	7.8	*239*	7.9	*1941*
	Total	7.2	*2024*	7.4	*1314*	7.5	*2089*	7.4	*1220*	7.5	*3630*
2014	Not well off	5.3^aw^	*135*	5.8^aw^	*32*	6.2^aw^	*183*	6.7^aw^	*60*	5.4^aw^	*185*
	Average	6.4^nw^	*554*	6.9^nw^	*210*	7.2^nw^	*558*	7.3^nw^	*876*	7.0^nw^	*984*
	Well off (ref)	7.1	*1939*	7.9	*645*	7.8	*1211*	7.8	*305*	7.9	*2053*
	Total	6.8	*2679*	7.5	*910*	7.5	*1960*	7.4	*1263*	7.5	*3275*
2018	Not well off	5.0^aw^	*48*	7.0	*21*	6.1^aw^	*63*				
	Average	6.4^nw^	*305*	6.7^w^	*116*	7.1^nw^	*223*				
	Well off (ref)	7.4	*1170*	7.8	*492*	7.7	*751*				
	Total	7.1	*1546*	7.5	*665*	7.5	*1061*	7.5	*762*	7.3	*2172*
**Linear regressions**		**B**	**95% C.I.**	**B**	**95% C.I.**	**B**	**95% C.I.**	**B**	**95% C.I.**	**B**	**95% C.I.**
	PFW (1–5)	0.65***	0.60 to 0.69	0.68***	0.62 to 0.73	0.48***	0.44 to 0.51	0.40***	0.32 to 0.48	0.68***	0.64 to 0.72
	Survey year	-0.03***	-0.03 to -0.02	0.02***	0.01 to 0.03	-0.01***	-0.02 to -0.01	-0.02***	-0.03 to -0.01	0.00	-0.01 to 0.02
	Girl	-0.65***	-0.72 to -0.57	-0.44***	-0.53 to -0.34	-0.27***	-0.34 to -0.21	-0.47***	-0.56 to -0.38	-0.26***	-0.34 to -0.19
	Adjusted R	0.132		0.109		0.083		0.042		0.114	
	F	446.3	0.000	235.2	0.000	255.4	0.000	77.0	0.000	355.2	0.000

In the descriptive statistics: Significant (*p* < 0.05) test results between PFW groups: ^w^= compared to the well off group ^a^=compared to the average group. ^n^=compared to the not well off group

In the regression models: * *p* < 0.05 ** *p* < 0.01 ***p < 0.001. *P*-values adjusted for multiple testing

The multiple linear regressions showed that there was a positive statistically significant association between PFW and LS among adolescents in all Nordic countries. The association varied among countries: from B = 0.40 in Denmark to B = 0.68 in both Norway and Iceland.

The regression analyses (Tables 3 and 4) also revealed that LS decreased over time among adolescents in Denmark, Sweden and Finland. In contrast, LS increased over time among adolescents in Norway. In all countries, LS was lower among girls than boys.

### The at-risk-of-poverty rate and the prevalence of subjective health complaints and levels of life satisfaction in the Nordic countries

The multilevel regression analyses showed that most of the variance in both SHC and LS occurred between individuals and that only small similarities were found among adolescents within countries (Table 5).

**Table 5:** SHC and LS among adolescents and the at-risk-of-poverty rate, all Nordic countries, 2002–2018

	SHC			LS		
	Model 1	Model 2	Model 3	Model 1	Model 2	Model 3
Survey year		0.01***	0.01***		0.00***	0.00***
*Individual-level variables*						
Gender			3.37***			-0.41***
Family affluence			0.01			0.07***
Perceived family wealth			-1.31***			0.55***
*Country-level variables*						
At-risk-of-poverty rate		0.13***	0.16***		-0.04***	-0.07***
ICC	4.4%	4.3%	8.0%	1.1%	0.8%	2.9%
ICC boys	3.6%	3.5%	6.3%	0.6%	0.5%	2.5%
ICC girls	6.0%	5.9%	9.4%	1.8%	1.5%	3.6%

*p < 0.05 **p < 0.01 ***p < 0.001

In the empty model, the intraclass correlation (ICC) for SHC was 0.044 (*p* = 0.080) for both boys and girls, 0.036 (*p* = 0.080) for boys and 0.060 (*p* = 0.080) for girls. This means that 4.4% of the individual variation in SHC occurred at the country level and might be attributable to contextual country-level factors. In model 2, the at-risk-of-poverty rate was positively related to SHC (B = 0.13, *p =* 0.000), and the ICC was 0.043 (*p* = 0.083). In model 3, where gender and individual-level SES variables were added, the relation remained positive (B = 0.16, *p* = 0.000), and the ICC increased to 0.08 (*p* = 0.085).

For LS, the ICC was 0.011 (*p* = 0.081) for both boys and girls and 0.006 (*p* = 0.088) for boys and 0.018 girls (*p* = 0.081) in the empty model. This means that 1.1% of the individual differences in LS occurred at the country level and might be attributable to contextual country-level factors. In the second model, the at-risk-of-poverty rate was negatively related to LS (B = -0.04, *p* = 0.000), and the ICC was 0.008 (*p* = 0.082). In the third model, the relation remained negative (B = -0.07, *p* = 0.000), and the ICC increased to 0.029 (*p* = 0.082).

## Discussion

The aims of the study were twofold. First, to examine absolute and relative socioeconomic inequalities in SHC and LS among adolescents in Nordic countries. Second, to explore whether SHC and LS were related to income inequality in terms of the at-risk-of-poverty rate at the country level.

Socioeconomic inequalities were found in both subjective health complaints and life satisfaction among adolescents in all Nordic countries from 2002 − 2018. Socioeconomic inequalities were similar among girls and boys in all countries except Denmark, where inequalities were more pronounced among girls in some cases. The findings in this study support both the absolute-income and relative-income hypotheses. The study also showed that the relation between perceived family wealth and adolescents’ mental health was stronger compared to family affluence, which indicates that relative socioeconomic conditions matters more than absolute socioeconomic conditions. The results corroborate previous research among adolescents in Sweden showing that subjective appraisals of SES are a stronger tool for identifying inequalities in health [37].

The results also showed that the higher the at-risk-of-poverty rate, the higher the prevalence of SHC and the lower the LS among adolescents. The findings are in line with the income-inequality hypothesis and with previous research using the Gini coefficient [21, 24, 26–28]. The increase of symptom load may be reflected in an increased demand for services in the future. Prospective studies have shown an association between health complaints in adolescence and later symptoms of depression and anxiety [38] and later diagnosis of depression and anxiety [39].

The present study adds to previous literature regarding probable causes behind the gradual increase in SHC among adolescents in Sweden [6–8], as the results suggest that increased income inequality has contributed to the increase in mental health problems. As mentioned in the introduction, there are of course other plausible factors behind the increase in Sweden, such as changes in the Swedish school system and in the labour market.

The present study also adds to the previous literature by using an alternative measure of income inequality (the at-risk-of-poverty rate) compared to most previous studies examining the association between income inequality and health, which have used Gini. The at-risk-of-poverty rate is a more straightforward measure of earned income across the population and does not take into account other types of income that may vary across years, such as capital income. However, the two measures are highly correlated [12]. The results suggest that the at-risk-of-poverty rate may be used as an alternative measure of income inequality, not least among countries where levels of income inequality in terms of the Gini may be similar.

### Strengths and limitations

Some countries have experienced declining response rates among schools and students, which may have affected the representativeness of the samples. One such reason may be the use of informed consent from parents in Norway in 2018. Nonparticipating students may have both lower SES and worse mental health than the participants, potentially affecting the results in this study (underestimating the socioeconomic differences in mental health, as well as levels of SHC and LS).

Another limitation is the use of the HBSC-SCL as a sum score (a continuous variable) instead of as a dichotomous variable (e.g. those with at least two health complaints, more than once a week). We have only examined mean values of health complaints and not looked into the distribution of health complaints among adolescents. However, a recent Swedish study has revealed that the increase in complaints among Swedish adolescents was greatest among adolescents who report frequent and co-occurring complaints [32].

Furthermore, the translation of the PFW item into Danish differs from the translations in the other Nordic countries. The wording in Danish is “Hvor rig er din familie?” (in English: How rich is your family?) with the response options: “meget rig”, “rig”, “som gennemsnittet”, “fattig” and “meget fattig” (in English: “very rich”, “rich”, “average”, “poor”, “very poor”). The translation may be one reason why the majority of Danish children answered “average”, in contrast to the other Nordic countries, where the majority answered “well off” or “very well off” and may partly explain why the results in this study are different for Denmark compared to the other Nordic countries.

Additionally, the at-risk-of-poverty rate measure has some limitations; for instance, it does not take into account the value of debt and property [9]. Therefore, it does not provide full information on families’ socioeconomic conditions.

Finally, we applied multilevel linear regression on a limited data sample including only five countries. This limits the strength of the analyses since the unbiassedness of the estimators is not assured [40, 41]. To some extent this limitation is compensated for by having a long time series (five data points per country except Iceland) and a large number of students (41,148). However, the estimates from the multilevel analyses have to be interpreted with some caution.

The study’s main strength is the use of a large dataset from the international HBSC study, which is carried out in accordance with a common research protocol in all 50 participating countries across Europe and Canada. The protocol covers all steps of the data collection, including the validation and translation of instruments, the design and piloting of the questionnaire, the sampling of schools and students and the cleaning and coding of collected data.

Another important strength is that the instruments used to measure socioeconomic conditions (the FAS and the PFW item) and health outcomes (HBSC-SCL and Cantril’s ladder) are well validated (please see [5] for a full description of the validation studies). The HBSC-SCL and Cantril’s ladder are widely used within research (please see the scoping review by Currie and Morgan, 2020 [42]).

## Conclusions

Socioeconomic inequalities in SHC and LS among adolescents persist in the 2000s and remain a key public health challenge in all Nordic countries. Sweden shows average socioeconomic inequalities from a Nordic perspective: the inequalities are larger in Iceland and Norway and smaller in Finland and Denmark. The at-risk-of-poverty rate is a probable contributor to higher levels of SHC and lower levels of LS in Sweden compared to the other Nordic countries.

The results demonstrate the continued need for policies that improve families’ absolute and relative socioeconomic conditions and reduce income inequality at the country level. Such policies are even more important in light of the prevailing economic recession in Europe in the postpandemic era, the climate crisis and the ongoing war in Ukraine.

### Electronic supplementary material

Below is the link to the electronic supplementary material.

Supplementary Material 1

Supplementary Material 2

Supplementary Material 3

Supplementary Material 4

## Data Availability

Data are available from the HBSC network. Visit https://www.uib.no/en/hbscdata for more information.
