# Supplementary material for: Socioeconomic inequalities in adolescent mental health in the Nordic countries in the 2000s - A study using cross-sectional data from the Health Behaviour in School-aged Children study
Source: Arch Public Health. 2024 Feb 7;82:20. doi: 10.1186/s13690-024-01240-5 (PMC10848422; doi:10.1186/s13690-024-01240-5)
Supplement: Supplementary file 1 — Supplementary Material 1 [file 13690_2024_1240_MOESM1_ESM.docx]

Supplementary Table 1 Number of boys and girls participating in the HBSC survey in each Nordic country, 2002−2018

|  |  | **Sweden** | | **Norway** | | **Finland** | | **Denmark** | | **Iceland** | |
| --- | --- | --- | --- | --- | --- | --- | --- | --- | --- | --- | --- |
|  |  | % | *n* | % | *n* | % | *n* | % | *n* | % | *n* |
| 2002 | Boys | 50.0% | *609* | 49.3% | *799* | 49.8% | *867* | 47.9% | *656* |  |  |
|  | Girls | 50.0% | *609* | 50.7% | *823* | 50.2% | *874* | 52.1% | *713* |  |  |
| 2006 | Boys | 49.3% | *752* | 53.3% | *818* | 46.9% | *790* | 49.1% | *762* | 50.3% | *947* |
|  | Girls | 50.7% | *774* | 46.7% | *716* | 53.1% | *895* | 50.9% | *790* | 49.7% | *936* |
| 2010 | Boys | 50.7% | *1059* | 53.1% | *711* | 47.8% | *1008* | 47.1% | *577* | 50.9% | *1873* |
|  | Girls | 49.3% | *1031* | 46.9% | *628* | 52.2% | *1102* | 52.9% | *649* | 49.1% | *1807* |
| 2014 | Boys | 49.1% | *1358* | 48.1% | *467* | 48.7% | *956* | 46.2% | *584* | 50.0% | *1659* |
|  | Girls | 50.9% | *1408* | 51.9% | *503* | 51.3% | *1009* | 53.8% | *679* | 50.0% | *1657* |
| 2018 | Boys | 48.3% | *771* | 48.3% | *327* | 49.3% | *531* | 51.2% | *392* | 49.3% | *1074* |
|  | Girls | 51.7% | *825* | 51.7% | *350* | 50.7% | *545* | 48.8% | *374* | 50.7% | *1104* |
