# Supplementary material for: Socioeconomic inequalities in adolescent mental health in the Nordic countries in the 2000s - A study using cross-sectional data from the Health Behaviour in School-aged Children study
Source: Arch Public Health. 2024 Feb 7;82:20. doi: 10.1186/s13690-024-01240-5 (PMC10848422; doi:10.1186/s13690-024-01240-5)
Supplement: Supplementary file 2 — Supplementary Material 2 [file 13690_2024_1240_MOESM2_ESM.docx]

Supplementary Table 2 FAS among 15-year-olds in the Nordic countries, 2002−2018

| Year |  | **Sweden** | | **Norway** | | **Finland** | | **Denmark** | | **Iceland** | |
| --- | --- | --- | --- | --- | --- | --- | --- | --- | --- | --- | --- |
| 2002 | Mean | 5.75 |  | 6.24 |  | 5.06 |  | 5.47 |  |  |  |
|  | Low | 24.3% | *294* | 15.7% | *252* | 17.8% | *304* | 19.3% | *260* |  |  |
|  | Medium | 59.4% | *719* | 60.5% | *973* | 61.4% | *1052* | 60.9% | *822* |  |  |
|  | High | 16.3% | *197* | 23.9% | *384* | 20.8% | *356* | 19.9% | *268* |  |  |
| 2006 | Mean | 6.17 |  | 6.80 |  | 5.39 |  | 6.11 |  | 6.82 |  |
|  | Low | 17.4% | *261* | 20.6% | *313* | 13.1% | *215* | 18.5% | *274* | 19.1% | *343* |
|  | Medium | 59.6% | *894* | 63.4% | *963* | 67.5% | *1111* | 58.8% | *873* | 64.7% | *1162* |
|  | High | 22.9% | *344* | 16.0% | *243* | 19.4% | *319* | 22.7% | *337* | 16.2% | *291* |
| 2010 | Mean | 6.36 |  | 7.28 |  | 6.07 |  | 6.73 |  | 7.13 |  |
|  | Low | 20.2% | *409* | 17.5% | *232* | 17.0% | *350* | 20.1% | *242* | 14.4% | *519* |
|  | Medium | 53.7% | *1087* | 57.8% | *768* | 63.6% | *1305* | 67.3% | *808* | 63.0% | *2265* |
|  | High | 26.1% | *529* | 24.7% | *328* | 19.4% | *398* | 12.6% | *151* | 22.6% | *813* |
| 2014 | Mean | 6.55 |  | 7.18 |  | 6.05 |  | 6.78 |  | 5.98 |  |
|  | Low | 17.2% | *457* | 15.7% | *144* | 13.6% | *261* | 16.0% | *193* | 25.5% | *761* |
|  | Medium | 57.2% | *1519* | 66.6% | *611* | 65.9% | *1262* | 69.9% | *843* | 52.4% | *1564* |
|  | High | 25.6% | *681* | 17.7% | *162* | 20.5% | *393* | 14.1% | *170* | 22.2% | *662* |
| 2018 | Mean | 6.67 |  | 7.15 |  | 6.41 |  | 6.78 |  | 6.71 |  |
|  | Low | 15.0% | *233* | 17.4% | *115* | 21.8% | *222* | 23.5% | *164* | 15.0% | *320* |
|  | Medium | 70.3% | *1091* | 65.8% | *434* | 59.3% | *604* | 58.5% | *408* | 61.2% | *1302* |
|  | High | 14.7% | *229* | 16.8% | *111* | 18.9% | *192* | 17.9% | *125* | 23.8% | *507* |
