# Supplementary material for: Socioeconomic inequalities in adolescent mental health in the Nordic countries in the 2000s - A study using cross-sectional data from the Health Behaviour in School-aged Children study
Source: Arch Public Health. 2024 Feb 7;82:20. doi: 10.1186/s13690-024-01240-5 (PMC10848422; doi:10.1186/s13690-024-01240-5)
Supplement: Supplementary file 3 — Supplementary Material 3 [file 13690_2024_1240_MOESM3_ESM.docx]

#### Supplementary Table 3 PFW among 15-year-olds in the Nordic countries, 2002−2018

| Year |  | **Sweden** | | **Norway** | | **Finland** | | **Denmark** | | **Iceland** | |
| --- | --- | --- | --- | --- | --- | --- | --- | --- | --- | --- | --- |
| 2002 | Mean, STD | 3.88 | 0.94 | 3.64 | 0.80 | 3.86 | 0.90 | 3.14 | 0.47 |  |  |
|  | Well off (%, *n*) | 65.1% | *786* | 56.8% | *916* | 60.5% | *1044* | 17.2% | *233* |  |  |
|  | Average (%, *n*) | 25.8% | *312* | 34.7% | *560* | 32.7% | *564* | 79.7% | *1081* |  |  |
|  | Not well off (%, *n*) | 9.1% | *110* | 8.4% | *136* | 6.8% | *118* | 3.1% | *42* |  |  |
| 2006 | Mean, STD | 4.06 | 0.88 | 4.03 | 0.75 | 3.97 | 0.88 | 3.20 | 0.53 | 3.96 | 0.86 |
|  | Well off (%, *n*) | 74.0% | *1121* | 69.0% | *1037* | 64.6% | *1079* | 23.0% | *345* | 68.2% | *1272* |
|  | Average (%, *n*) | 19.3% | *292* | 25.8% | *388* | 31.1% | *519* | 73.3% | *1101* | 27.1% | *506* |
|  | Not well off (%, *n*) | 6.7% | *101* | 5.1% | *77* | 4.3% | *72* | 3.7% | *56* | 4.6% | *86* |
| 2010 | Mean, STD | 4.08 | 0.91 | 3.86 | 0.85 | 3.96 | 0.90 | 3.17 | 0.53 | 3.87 | 0.89 |
|  | Well off (%, *n*) | 73.1% | *1489* | 69.0% | *815* | 64.3% | *1345* | 20.2% | *241* | 60.9% | *1960* |
|  | Average (%, *n*) | 21.3% | *433* | 25.0% | *295* | 28.6% | *599* | 76.2% | *910* | 32.4% | *1042* |
|  | Not well off (%, *n*) | 5.6% | *115* | 6.0% | *71* | 7.1% | *149* | 3.7% | *44* | 6.8% | *218* |
| 2014 | Mean, STD | 4.05 | 0.86 | 3.90 | 0.77 | 3.87 | 0.93 | 3.21 | 0.60 | 3.93 | 0.89 |
|  | Well off (%, *n*) | 73.7% | *1988* | 72.7% | *647* | 62.0% | *1211* | 24.6% | *305* | 63.5% | *2069* |
|  | Average (%, *n*) | 21.0% | *566* | 23.6% | *210* | 28.6% | *559* | 70.6% | *876* | 30.7% | *1001* |
|  | Not well off (%, *n*) | 5.3% | *144* | 3.7% | *33* | 9.4% | *183* | 4.8% | *60* | 5.8% | *189* |
| 2018 | Mean, STD | 4.18 | 0.83 | 4.03 | 0.75 | 4.06 | 0.91 |  |  |  |  |
|  | Well off (%, *n*) | 76.7% | *1202* | 78.0% | *494* | 72.3% | *758* |  |  |  |  |
|  | Average (%, *n*) | 20.1% | *315* | 18.5% | *117* | 21.7% | *227* |  |  |  |  |
|  | Not well off (%, *n*) | 3.2% | *50* | 3.5% | *22* | 6.0% | *63* |  |  |  |  |
