# Supplementary material for: Socioeconomic inequalities in adolescent mental health in the Nordic countries in the 2000s - A study using cross-sectional data from the Health Behaviour in School-aged Children study
Source: Arch Public Health. 2024 Feb 7;82:20. doi: 10.1186/s13690-024-01240-5 (PMC10848422; doi:10.1186/s13690-024-01240-5)
Supplement: Supplementary file 4 — Supplementary Material 4 [file 13690_2024_1240_MOESM4_ESM.docx]

#### Supplementary Table 4 At-risk-of-poverty rate (all ages) in the Nordic countries 2001/02-2017/18

| Year | **Sweden** | **Norway** | **Finland** | **Denmark** | **Iceland** |
| --- | --- | --- | --- | --- | --- |
| 2001/02 | 10.0 | 10.5 | 11.0 | 10.0 |  |
| 2005/06 | 10.9 | 11.7 | 12.2 | 11.8 | 9.7 |
| 2009/10 | 14.6 | 11.5 | 13.5 | 13.2 | 10.0 |
| 2013/14 | 15.8 | 10.9 | 12.3 | 12.0 | 8.6 |
| 2017/18 | 16.1 | 12.6 | 11.8 | 12.6 | 9.5 |

Source: Eurostat <https://ec.europa.eu/eurostat/databrowser/view/ILC_LI02__custom_1367219/default/table?lang=en>
